# Ultrasound- and Molecular Sieves-Assisted Synthesis, Molecular Docking and Antifungal Evaluation of 5-(4-(Benzyloxy)-substituted phenyl)-3-((phenylamino)methyl)-1,3,4-oxadiazole-2(3*H*)-thiones

**DOI:** 10.3390/molecules21050484

**Published:** 2016-05-10

**Authors:** Urja D. Nimbalkar, Santosh G. Tupe, Julio A. Seijas Vazquez, Firoz A. Kalam Khan, Jaiprakash N. Sangshetti, Anna Pratima G. Nikalje

**Affiliations:** 1 Maulana Azad P. G. and Research Centre, Dr. Rafiq Zakaria Campus, Rauza Baug, Aurangabad 431001, India; urjasatish@gmail.com; 2 Biochemical Sciences Division, CSIR-National Chemical Laboratory, Dr. Homi Bhabha Road, Pashan, Pune 411008, India; sg.tupe@ncl.res.in; 3 Departamento de Química Orgánica, Facultad de Ciencias, Universidad of Santiago De Compostela, Alfonso X el Sabio, Lugo 27002, Spain; julioa.seijas@usc.es; 4 Y. B. Chavan College of Pharmacy, Dr. Rafiq Zakaria Campus, Rauza Baug, Aurangabad 431001, India; firozakhan05@gmail.com (F.A.K.K.); jnsangshetti@rediffmail.com (J.N.S.)

**Keywords:** 1,3,4-oxadiazoles, ultrasound, Mannich reaction, molecular sieves, antifungal activity, molecular docking

## Abstract

A novel series of 5-(4-(benzyloxy)substituted phenyl)-3-((phenyl amino)methyl)-1,3,4-oxadiazole-2(3*H*)-thione Mannich bases **6a**–**o** were synthesized in good yield from the key compound 5-(4-(benzyloxy)phenyl)-1,3,4-oxadiazole-2(3*H*)-thione by aminomethylation with paraformaldehyde and substituted amines using molecular sieves and sonication as green chemistry tools. The antifungal activity of the new products was evaluated against seven human pathogenic fungal strains, namely, *Candida albicans* ATCC 24433, *Candida albicans* ATCC 10231, *Candida glabrata* NCYC 388, *Cryptococcus neoformans* ATCC 34664, *Cryptococcus neoformans* PRL 518, *Aspergillus fumigatus* NCIM 902 and *Aspergillus niger* ATCC 10578. The synthesized compounds **6d**, **6f**, **6g**, **6h** and **6j** exhibited promising antifungal activity against the tested fungal pathogens. In molecular docking studies, derivatives **6c**, **6f** and **6i** showed good binding at the active site of *C. albicans* cytochrome P450 enzyme lanosterol 14 α-demethylase. The *in vitro* antifungal activity results and docking studies indicated that the synthesized compounds have potential antifungal activity and can be further optimized as privileged scaffolds to design and develop potent antifungal drugs.

## 1. Introduction

Mortality due to secondary fungal infections in immunocompromised patients such as patients with AIDS, autoimmune diseases, burns, radiotherapy or chemotherapy is becoming a serious problem worldwide, with 1.5 to 2 million deaths every year [1]. Most of the mortality is caused by species belonging to four genera, namely *Candida*, *Aspergillus*, *Cryptococcus* and *Pneumocystis*. Emerging fungal pathogens and development of resistance in pathogenic fungi to currently used antifungal drugs has further aggravated the situation [2,3]. Currently, a limited repertoire of drugs from five classes, namely polyenes, azoles, echinocandins, allylamines and fluoropyrimidines are used for the treatment of fungal infections. Apart from drug resistance in pathogens, drawbacks of existing drugs such as acute and chronic side-effects, less clinical efficiency, and effects on non-targeted cells has forced researchers around the world to search for novel and efficient antifungal drugs [2].

Heterocycles containing nitrogen and oxygen atoms are considered an important class of compounds in medicinal chemistry because of their versatile biological applications. The 1,3,4-oxadiazole scaffold is associated with diverse biological activities such as antifungal [4,5,6] antibacterial [7,8], antimycobacterial [9], anti-HIV [10], anti-hepatitis B virus [11], anticancer [12], anticonvulsant [13], anti-inflammatory [14], anti-malarial [15] and analgesic effects [16]. 5-Substituted-1,3,4-oxadiazole-2-thiones, which feature an exocylic sulphur at C-2 of a 1,3,4-oxadiazole skeleton represent an important type of compound class in the field of coordination chemistry because of their potential multifunctional donor sites, *viz* either exocyclic sulfur or endocyclic nitrogen [17] and known to possess CNS depressant [18] and tyrosinase inhibition [19] activity. 1,3,4-Oxadiazole-2-thiones belong to the class of five-membered aromatic *N*-heterocycles substrates with the N–H reactive center involved in tautomerism with S–H [20]. The three-component condensation of a compound containing an acidic hydrogen atom with substituted primary or secondary amines and an aldehyde is known as a Mannich reaction and the product of this reaction has reported to be of significance in the preparation of natural and synthetic molecules with a remarkable biological activity. The literature survey on Mannich reaction involving oxadiazole thiones has shown that these compounds undergo either *N*-aminomethylation or *S*-aminomethylation depending on the reaction conditions [21]. The multipurpose usage of the Mannich bases in pharmaceutical chemistry [22,23] prompted us to prepare a new series of 5-(4-(benzyloxy)substitutedphenyl)-3-((phenyl amino)methyl)-1,3,4-oxadiazole-2(3*H*)-thione based aminomethyl derivatives.

Ultrasound-assisted organic synthesis is a green synthetic approach and a powerful technique for the enhancement of reaction rates and yields [24,25]. Ultrasonic irradiation is enhanced due to the formation of high energy intermediates. It offers the possibility of accomplishing potential reactions in short time cycles with cheaper reagents and less extreme physical conditions [26,27]. It can also be considered as an important tool for conservation of energy and minimization of waste as compared to the conventional techniques [28]. Microporous 3Å molecular sieves are aluminosilicate minerals with chemical composition of 2/3K_2_O·1/3Na_2_O·Al_2_O_3_·2SiO_2_·9/2H_2_O. Since the 1990s, these molecular sieves have attracted considerable attention due to their potential use in catalysis [29]. Mannich reactions by conventional methods take 15 to 20 h for the synthesis of the same derivatives, whereas using ultrasound the reaction time is reduced to 1 to 2 h. In the present study we report the use of ultrasound- and molecular sieves-assisted Mannich reactions for the synthesis of 5-substituted 1,3,4-oxadiazole-2-thiones with primary and secondary amines. Molecular docking of the synthesized compounds and screening for antifungal potential against different yeast and filamentous fungal pathogens is also reported.

## 2. Results and Discussion

### 2.1. Chemistry

**Scheme 1:**
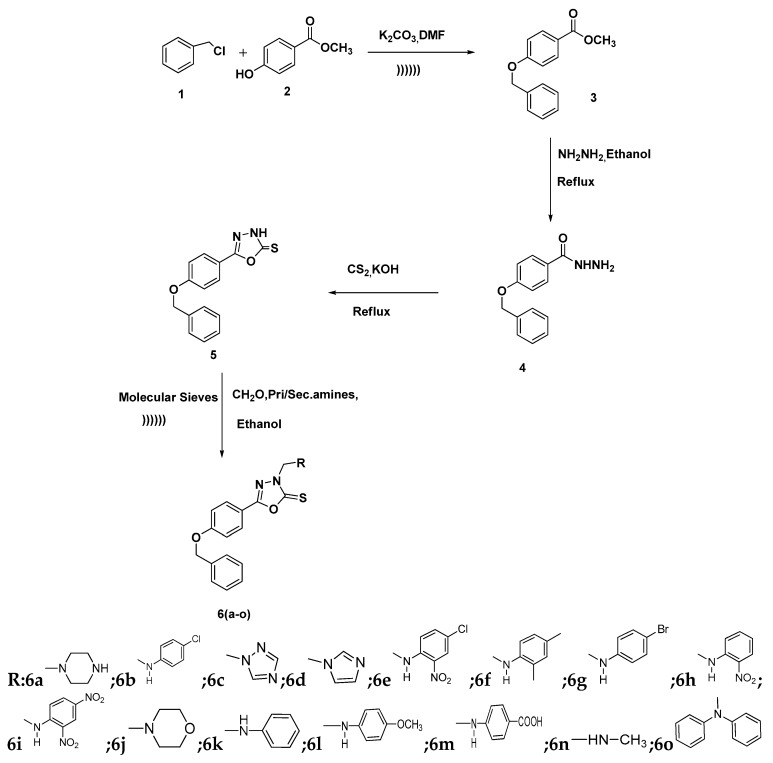
Synthesis of Mannich bases **6a**–**o**.

All the final compounds **6a**–**o** were synthesized following the procedure depicted in Scheme 1. The starting material methyl 4-(benzyloxy) benzoate (**3**) was synthesized by the reaction of methyl 4-hydroxybenzoate (**2**) and benzyl chloride(**1**) in K_2_CO_3_ and DMF as solvent in an ultrasonic processor for up to 4 h. Synthesis by a conventional method like stirring at room temperature required 20–28 h and by reflux nearly 8–10 h. The compound **3** obtained in good yield in step I is next refluxed with NH_2_NH_2_ to get 4-(benzyloxy) benzohydrazide (**4**). The reaction of the acid hydrazide **4** with carbon disulphide under basic conditions using KOH yielded 5-(4-(benzyloxy)phenyl)-1,3,4-oxadiazole-2(3*H*)-thione **5** [30]. *N*-Amino-methylation of (**5**) with various substituted primary amines and secondary amines and paraformaldehyde in presence of activated molecular sieves in ultrasonic processor under the Mannich reaction gave **6a**–**o** with good yields within 1–2 h (Table 1). Characterization data of the synthesized derivatives is presented in Table S1 (Supplementary Materials).

The structure of intermediate 5-(4-(benzyloxy)phenyl)-1,3,4-oxadiazole-2(3*H*)-thione (**5**) was confirmed by spectroscopic analysis. The IR spectrum showed bands at 3235 (NH), 1621 (C=N), 1596 (C–C), 1425 (C=S), 1258 and 1093 cm^−1^ (C–O–C). The ^1^H-NMR spectrum displayed a singlet at 3.8 ppm, assigned to the NH/SH tautomeric proton, a singlet at 5.17 ppm, integrating to 2H and assigned to the C–CH_2_–O group and a 9H multiplet at 7.0–7.9 for the two aromatic rings. In the ^13^C-NMR the –C=S carbon appeared at δ 189.9, along with the characteristic signals of the remaining carbon atoms. The HRMS mass spectrum showed (M + 1) molecular ion peak at *m*/*z* 285, in agreement with its molecular formula, C_15_H_12_O_2_N_2_S.

In the IR spectrum of compound **6j**, the aromatic C–H stretching vibration was observed at 3047 cm^−1^. The absorption band due to the CH_2_ groups of the morpholine moiety was seen at 2916/2850 cm^−1^. The C=N and C=S moieties showed their characteristic absorption bands at 1681 and 1356 cm^−1^, respectively. In ^1^H-NMR spectrum, the eight protons of the morpholine moiety resonated as two triplets at δ 2.75 and δ 3.62, while two singlet were seen at δ 5.02 and δ 5.16 for the N–CH_2_–N and –OCH_2_ protons, along with a multiplet at δ 7.06–8.02 for the nine aromatic ring protons. In the ^13^C-NMR spectrum, the signals observed at δ 67.91 and 70.12 were assigned to C2, C6 and C3, C5 of the morpholine ring, respectively. The signal due to the aryloxy methylene carbon appeared at δ 68.23. The aryloxymethine carbon atom resonated at δ 156.69, and the C=S carbon appeared at 177.14, while other aromatic carbon atoms appeared at δ 115.47, 119.51, 125.68, 128.17, 128.43, 128.61, 129.00, 131.97 and 135.64. Further, the structure of **6j** was also confirmed by recording its mass spectrum, which showed a molecular ion peak at *m*/*z* 383, in agreement with the assigned molecular formula, C_20_H_21_N_3_O_3_S.

### 2.2. In Vitro Antifungal Activity

The newly synthesized compounds **6a**–**o** were screened for *in vitro* antifungal activity against different yeast and filamentous fungal pathogens and were found to be fungistatic. All the compounds exhibited moderate antifungal activity against the tested yeast pathogens, *viz. Candida* and *Cryptococcus* strains, as compared to the standard fluconazole, the results are exhibited in Table 2. Derivatives **6i** and **6e** exhibited better antifungal activity than fluconazole against the filamentous *Aspergillus fumigatus* and *Aspergillus niger*, respectively. The antifungal activity exhibited by these compounds may be mainly due to the presence of various electron withdrawing groups such as bromine at the phenyl *para*-position for **6g** (4 μg/mL), imidazole for **6d** (6 μg/mL), morpholine for **6j** (8 μg/mL), and triazole in case of **6c** (11.2 μg/mL). Compound **6f** (5.4 μg/mL) with *ortho* and *para*-disubstituted toluyl and **6e** (24.5 μg/mL) showed good activity against *Candida glabrata* NCYC 388. The antifungal susceptibility testing results indicated that 5-(4-(benzyloxy) substituted phenyl)-3-((phenylamino)methyl)-1,3,4-oxadiazole-2(3*H*)-thione derivatives can act as a good scaffold to develop lead molecules with potent antifungal activity. The final derivatives possessing electron withdrawing groups on the phenyl ring at position 3 of the nitrogen of the 1,3,4-oxadiazole-2-thione ring acted as more potent compounds and exhibited excellent antifungal activity.

### 2.3. Molecular Docking Study

The azole class of antifungal drugs inhibits the cytochrome P450 lanosterol 14α-demethylase enzyme in the ergosterol synthesis pathway. As ergosterol is the major sterol component of the fungal cell membrane, inhibition of this enzyme and subsequent ergosterol depletion by the compounds leads to loss in fungal cell integrity and function [29]. Therefore, we carried out molecular docking of our oxadiazole thiones **6a**–**o** and the standard drug fluconazole into the active site of cytochrome P450 lanosterol 14α-demethylase of *Candida albicans*, using the VLife MDS 4.3 software, to understand the binding interactions. The binding energy and hydrogen bond interactions are presented in Table 3.

**Figure 1:**
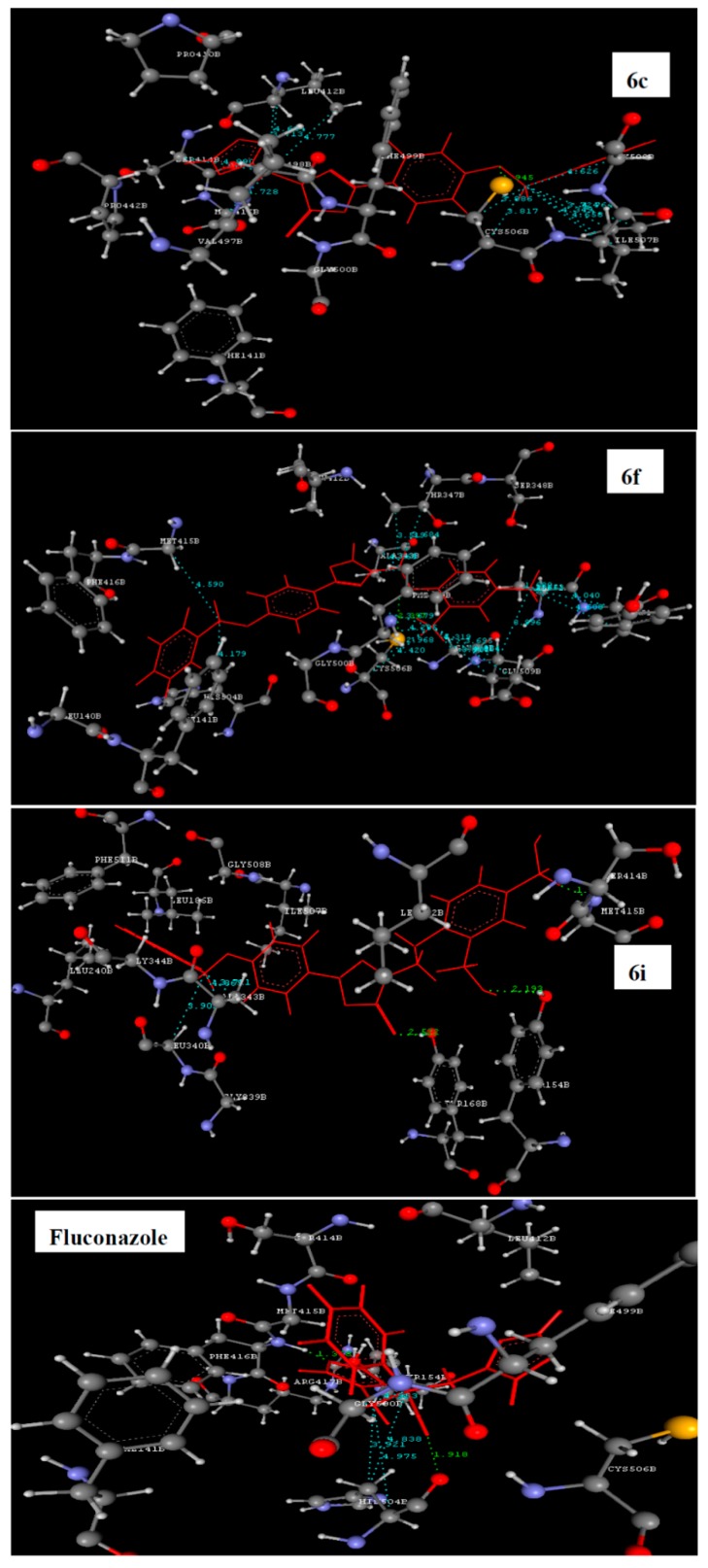
Docking of compounds **6c**, **6f**, **6i** and standard fluconazole. Ligands are shown in red color. Hydrogen bonds are shown in green color. Hydrophobic bonds are shown in sky blue color.

The most active synthesized compounds **6c**, **6f** and **6i** showed the lowest interaction energy, *i.e.*, −57.85, −65–25 and −61.03 kcal/mol, respectively. The standard drug fluconazole also showed good interaction energy (−67.29 kcal/mol). The docking results indicated that compounds were held in the active pocket by combination of various hydrogen and hydrophobic interactions with cytochrome P450 lanosterol 14α-demethylase. Diverse hydrophobic interactions occurred between the synthesized compounds and the active site chains of LEU186, LEU240, LEU340, ALA343, GLY344, THR347, LEU412, MET415, VAL440, PRO442, VAL497, PHE499, GLY500, HIS504, CYS506, ILE507, GLY508, GLU509, PHE511, ALA512 and TYR513. The amino acid residues such as TYR154, TYR168, LEU412, MET415, VAL497, GLY500 and CYS506 formed hydrogen bonds with the synthesized compounds. The docking interactions of the most active compounds **6c**, **6f** and **6i** are shown in Figure 1. The amino acid CYS506 formed hydrogen bonding (1.95 Å) with the –O–CH_2_–oxygen of the synthesized compound **6c**. The 1,2,4-triazole ring of compound **6c** was held in the active site by forming Vander Waal’s interactions with amino acid residues like PRO410, LEU412, PRO442, VAL497 and PRO498. The amino acid residue CYS506 (2.40 Å) formed a hydrogen bond with the –NH–nitrogen of compound **6f**. The amino acids TYR154 (2.19 Å), TYR168 (2.52 Å) and MET415 (2.82 Å) all formed hydrogen bonds with the 2-NO_2_ oxygen, the carbonyl oxygen and the 4-NO_2_ nitrogen, respectively, of compound **6i**. On the basis of the antifungal activity and docking results, it was found that compounds **6c**, **6f** and **6i** had potential to inhibit cytochrome P450 lanosterol 14α-demethylase of *C. albicans*.

## 3. Materials and Methods

### 3.1. General Information

All the chemicals used for synthesis were procured from Merck (Mumbai, Maharashtra, India), Sigma (Mumbai), HiMedia (Mumbai) or Qualigens (Mumbai) and used without further purification. The progress of each reaction was monitored by ascending thin layer chromatography (TLC) using pre-coated silica gel F254 aluminum TLC sheets (Merck) and the spots were visualized by UV light and iodine vapors. Elemental analyses (C, H, and N) were done with a FLASHEA 112 Shimadzu’ analyzer (Mumbai) and all analyses were consistent (within 0.4%) with theoretical values. Infrared (IR) spectra were recorded on a PS 4000 FTIR (JASCO, Tokyo, Japan) using KBr pellets. ^1^H- and ^13^C-NMR (200 MHz) spectra were recorded on a ACF 200 spectrometer (Bruker, Billerica, MA, USA) fitted with an Aspect 3000 computer and all the chemical shifts (ppm) were referred to internal TMS for 1H and chloroform-d for^13^C-NMR. ^1^H-NMR data are reported in the order of chemical shift, multiplicity (s, singlet; d, doublet; t, triplet; q, quartet; br, broad; br s, broad singlet; m, multiplet and/ or multiple resonance), number of protons. A Micro TOF-Q-II (Bruker Daltonics, Billerica, MA, USA with electron spray ionization (ESI) was used to obtain the HRMS data. For ultrasound irradiation Vibra cell VCX-500 with solid probe was used (Sonics, Newtown, CT, USA).

### 3.2. Synthesis of Methyl-4-(Benzyloxy)benzoate *(**3**)*

For the synthesis of methyl-4-(benzyloxy)benzoate, the reaction between equal 0.01 molamounts of methyl-4-hydroxybenzoate and chloromethylbenzene in *N*,*N*-dimethylformamide (DMF) as solvent usingK_2_CO_3_ as mild base was carried with under an ultrasonic processor at room temperature for up to 4 h. The solution was then poured into ice-water. The solid obtained was filtered and recrystallized from ethanol. Colour: white; m.p. 105 °C (100 °C reported in chemspider)

### 3.3. Synthesis of 4-(Benzyloxy)benzohydrazide *(**4**)*

For the synthesis of the substituted benzohydrazide, a mixture of the corresponding ester (20 mmol), 85% hydrazine hydrate (20 mmol) in ethanol (35 mL) was refluxed for 6 h. After that, the solution was poured into ice-water. The solid was filtered and recrystallized from ethanol. Colour: white; m.p.138 °C (140 °C reported in chemspider)

### 3.4. Synthesis of 5-(4-(Benzyloxy)phenyl)-1,3,4-oxadiazole-2(3H)-thione

Equimolar quantities of the substituted benzohydrazide (5 mmol) and potassium hydroxide (5 mmol) were dissolved in 95% ethanol (20 mL). The mixture was allowed to stir for several minutes at room temperature and then carbon disulfide (15 mmol) was slowly added dropwise to the reaction system and the mixture was heated to reflux. The residue obtained was dissolved in water (50 mL) and diluted hydrochloric acid was added to adjust the pH value of the solution to 5–6. The precipitate was collected washed with water for several times and dried and recrystallized from ethanol. Colour: white; m.p. 160 °C.

### 3.5. General Procedure for the Synthesis of 5-(4-(Benzyloxy)substituted phenyl)-3-((phenylamino)methyl)-1,3,4-oxadiazole-2(3H)-thiones ***6a**–**o***

To a methanolic solution of 5-(4-(benzyloxy) phenyl)-1,3,4-oxadiazole 2(3*H*) thione (**5**), (10 mmol), paraformaldehyde (15 mmol) and a substituted primary/secondary amine (10 mmol) in methanol 25 mL were added with constant stirring. Preactivated molecular sieves (3Å) were also added to the reaction mixture to absorb the water formed. The resulting mixture was subjected to ultrasonic irradiation for 1–2 h at room temperature. The precipitated solids were filtered, washed with ice water. The progress of the reaction was monitored by TLC using 1:4 ethyl acetate–*n*-hexane as a solvent system. The reaction was quenched with crushed ice and the solid obtained was recrystallized from methanol to yield the title compounds **6**(**a–o**) mentioned in Table 1 (see also Table S1).

*5-(4-(Benzyloxy)phenyl)-1,3,4-oxadiazole-2(3H)-thione* (**5**): Recrystallized from ethanol; yield: 90%; m. p.: 160 °C. IR (KBr) ν_max_ (cm^−1^): 3235 (NH), 3100–3000 aromatic CH stretch,2870–2800 aliphatic CH stretch, 1621 (C=N), 1596 (C–C), 1250 (C=S), 1258 and 1093 (C–O–C), ^1^H-NMR (CDCl_3_), δ ppm: 3.8 (s, 1H, NH), 5.17 (s, 2H, C–CH_2_–O), 7.07.9 (m, 9H, two aromatic ring), ^13^C-NMR (CDCl_3_, δ ppm): 70.8, 114.4, 116.3, 127.1, 127.6, 128.9, 130.2, 136.7, 157.0, 161.3, 189.9, *m*/*z*: 284.1 (100.0%), 285.1 (18.0%), 286.1 (6.5%), Molecular Formula: C_15_H_12_N_2_O_2_S. Elemental Analysis: Calculated: (C, H, N) 63.3, 4.25, 9.85 Found: 63.5, 4.22, 9.87.

*5-(4-(Benzyloxy)phenyl)-3-(piperazine-1-ylmethyl)-1*,*3*,*4-oxadiazole-2(3H)-thione* (**6a**): Yield: 80%; m.p.: 205 °C. IR (KBr) ν_max_ (cm^−1^): 3245 (NH), 3100–3000 aromatic CH stretch, 2870–2800 aliphatic CH stretch, 1623 (C=N), 1598 (C–C), 1252 (C=S), 1260 and 1095 (C–O–C), 1280 (C-N sec. amine piperazine), 1560 (N–H sec. amine piperazine), ^1^H-NMR (CDCl_3_), δ ppm: 1.91 (s, 1H, –NH piperazine), 2.37 (t, 4H, N–CH_2_–C piperazine), 2.65 (t, 4H, C–CH_2_–NH piperazine), 3.72 (s, 2H, N–CH_2_–N), 5.16 (s, 2H, C–CH_2_–O), 7.38–8.02 (m, 9H, aromatic ring), ^13^C-NMR ( CDCl_3_, δ ppm): 45.91, 54.7, 70.8, 71.0, 114.4, 116.3, 127.1, 127.5, 128.9, 130.2, 136.7, 157.0, 163.3, 177.1., *m*/*z* 382.15 (100.0%), 383.15 (22.8%), 384.14 (4.5%), Molecular Formula: C_20_H_22_N_4_O_2_S. Elemental Analysis: Calculated (C, H, N) 62.80, 5.80, 14.65, Found: 62.76, 5.74, 14.62.

*5-(4-(Benzyloxy)phenyl)-3-(((4-chlorophenyl)amino)methyl)-1*,*3*,*4-oxadiazole-2(3H)-thione* (**6b**): Yield: 81%; m.p.: 180 °C. IR (KBr) ν_max_ (cm^−1^):3230 (NH), 3100–3000 aromatic CH stretch, 2870–2800, aliphatic CH stretch, 1625 (C=N), 1592 (C–C), 1235 (C=S), 1261 and 1094 (C–O–C), 3470 (N–H primary amine) 744 (C–Cl), ^1^H-NMR (CDCl_3_), δ ppm: 4.0 (S, 1H, –NH), 4.42 (S, 2H, N–CH_2_–N–), 5.16 (S, 2H, –OCH_2_), 6.54 and 8.02 (m,13H, aromatic rings), ^13^C-NMR (CDCl_3_, δ ppm): 70.8, 114.4, 114.9, 116.3, 121.1, 127.1, 127.6, 128.9, 129.6, 130.2, 136.7, 145.7, 157.0, 161.3, 177.1., *m*/*z* 423.08 (100.0%), 425.08 (36.9%), 424.08 (25.7%), Molecular Formula: C_22_H_18_ClN_3_O_2_S. Elemental Analysis: Calculated (C, H, N) 62.33, 4.28, 9.91, Found: 62.28, 4.22, 9.88.

*3-((1H-1*,*2*,*4-Triazol-1-yl)methyl) 5-(4-(benzyloxy)phenyl)-1*,*3*,*4-oxadiazole-2(3H)-thione* (**6c**): Yield: 78%; m.p.: 235 °C. IR (KBr) ν_max_ (cm^−1^): 3100–3000 aromatic CH stretch, 2870–2800, aliphatic CH stretch, 1621 (C=N), 1596 (C–C), 1425 (C=S), 1258 and 1093 (C–O–C), 1290 (C–N), 1622 (C=N triazole), ^1^H-NMR (CDCl_3_), δ ppm: 5.09 (s, 2H, N–CH_2_–N), 5.16 (S, 2H, –OCH_2_),7.06–8.02 (m, 9H, aromatic rings), 8.05 and 8.68 (s, 1H, NH triazole ring), ^13^C-NMR (CDCl_3_, δ ppm): 70.8, 73.6, 114.4, 116.3, 127.1, 127.6, 128.9, 130.2, 136.7, 143.8, 151.5, 157.0, 161.3, 177.1, *m*/*z* 365.09 (100.0%), 366.10 (19.7%), 367.09 (4.5%), Molecular Formula: C_18_H_15_N_5_O_2_S; Elemental Analysis: Calculated (C, H, N) 59.16, 4.14, 19.17, Found: 59.11, 4.10, 19.11.

*3-((1H-Imidazol-1-yl)methyl) 5-(4-(benzyloxyphenyl)-1*,*3*,*4-oxadiazole-2(3H)-thione* (**6d**): Yield: 79%; m.p.: 240 °C. IR (KBr) ν_max_ (cm^−1^): 3100–3000 aromatic CH stretch, 2870–2800 aliphatic CH stretch, 1621 (C=N), 1596 (C–C), 1425 (C=S), 1258 and 1093 (C–O–C), 1290 (C–N), 1622 (C=N imidazole), 1625 (C=C imidazole ring), ^1^H-NMR (CDCl_3_), δ ppm: 5.09 (s, 2H, N–CH_2_–N), 5.16 (S, 2H, –OCH_2_), 6.77, 7.13 and 7.83 (s, 1H each of imidazole ring), 7.06–8.02 (m, 9H, aromatic rings), ^13^C-NMR (CDCl_3_, δ ppm): 70.8, 71.7, 114.4, 116.3, 120.6, 127.1, 127.6, 128.1, 128.9, 130.2, 136.7, 137.8, 157.0, 161.3, 177.1, *m*/*z* 364.10 (100.0%), 365.10 (22.9%), 366.10 (5.4%), Molecular Formula: C_19_H_16_N_4_O_2_. Elemental Analysis: Calculated (C, H, N) 62.62, 4.43, 15.37 Found: 62.58, 4.40, 15.34.

*5-(4-(Benzyloxy)phenyl)-3-(((4-chloro-2-nitrophenyl)amino)methyl)-1*,*3*,*4-oxadiazole-2(3H)-thione* (**6e**): Yield: 81%; m.p.: 218 °C. IR (KBr) ν_max_ (cm^−1^): 3549(NH), 3100–3000 aromatic CH stretch, 2870–2800 aliphatic CH stretch, 1625 (C=N), 1590 (C–C), 1526 and 1346 (NO_2_), 1429 (C=S), 1263 and 1089 (C–O–C), 1490 (C–NO_2_), 744 (C–Cl); ^1^H-NMR (DMSO-*d*_6_), δ ppm: 4.0 (S, 1H, –NH), 4.42 (S, 2H, N–CH_2_–N–), 5.16 (S, 2H, –OCH_2_), 7.06–8.13 (m, 12H, aromatic rings). ^13^C-NMR (CDCl_3_, δ ppm): 69.8, 70.8, 114.4, 115.8, 116.3, 122.0, 127.1, 127.6, 128.9, 130.2, 131.5, 135.7, 136.7, 144.8, 157.0, 161.3, 177.1, *m*/*z* 468.07 (100.0%), 470.06 (36.5%), 469.07 (24.9%), Molecular Formula: C_22_H_17_ClN_4_O_4_S. Elemental Analysis: Calculated (C, H, N) 56.35, 3.65, 11.95, Found 56.30, 3.61, 11.92.

*5-(4-(Benzyloxy)phenyl)-3-(((2*,*4-dimethylphenyl)amino)methyl)-1*,*3*,*4-oxadiazole-2(3H)-thione* (**6f**): Yield: 79%; mp: 230 °C. IR (KBr) νmax (cm^−1^): 3230 (NH), 3100–3000 aromatic CH stretch, 2870–2800 aliphatic CH stretch, 1616 (C=N), 1595 (C–C), 1427 (C=S), 1252 and 1151 (C–O–C), 1460 (Aromatic C–CH_3_), ^1^H-NMR (CDCl_3_), δ ppm: 2.10 (s, 3H, –CH_3_), 2.30 (s, 3H, –CH_3_), 4.0 (S, 1H, –NH), 4.42 (S, 2H, N–CH_2_–N–), 5.16 (S, 2H, –OCH_2_),6.36–8.02 (m, 12H, aromatic rings),^13^C-NMR (CDCl_3_, δ ppm): 17.9, 21.6, 70.8, 71.1, 113.3, 114.4, 116.3, 126.8, 127.1, 127.6, 128.9, 130.2, 131.7, 136.2, 136.7, 143.5, 157.0, 161.3, 177.1 *m*/*z* 417.15 (100.0%), 418.15 (27.9%), 419.15 (5.0%) Molecular Formula: C_24_H_23_N_3_O_2_S. Elemental Analysis: Calculated (C, H, N) 69.04, 5.55, 10.06, Found: 69.00, 5.51, 10.02.

*5-(4-(Benzyloxy)phenyl)-3-(((4-bromophenyl)amino)methyl)-1*,*3*,*4-oxadiazole-2(3H)-thione* (**6g**): Yield: 81%; m.p.: 228 °C. IR (KBr) ν_max_ (cm^−1^): 3235 (NH), 3100–3000 aromatic CH stretch, 2870–2800 aliphatic CH stretch, 1621 (C=N), 1593 (C–C), 1424 (C=S), 1260 and 1123 (C–O–C), 690 (C–Br), ^1^H-NMR (DMSO-*d*_6_), δ ppm: 4.0 (S, 1H, –NH), 4.42 (S, 2H, N–CH_2_–N–), 5.16 (S, 2H, –OCH_2_), 6.59–8.02 (m, 13H, aromatic rings), ^13^C-NMR ( CDCl_3_ ,δ ppm): 70.8, 114.4, 114.5, 115.1, 116.3, 127.1, 127.6, 128.9, 130.2, 132.4, 136.7, 146.6, 157.0, 161.3, 177.1, *m*/*z* 469 (100%), 467 (97.4%), 470 (25.7%), 471.2 (4.3%). Molecular Formula: C_22_H_18_BrN_3_O_2_S. Elemental Analysis: Calculated (C, H, N) 56.42, 3.87, 8.97, Found: 56.38, 3.84, 8.93.

*5-(4-(Benzyloxy)phenyl)-3-(((2-nitrophenyl)amino)methyl)-1*,*3*,*4-oxadiazole-2(3H)-thione* (**6h**): Yield: 90%; m.p.: 220 °C.IR (KBr) ν_max_ (cm^−1^): 3245 (NH), 3100–3000 aromatic CH stretch, 2870–2800 aliphatic CH stretch, 1622 (C=N), 1590 (C–C), 1522 and 1345 (NO_2_), 1427 (C=S), 1261 and 1088 (C–O–C), 1401 (C–NO_2_), 755; ^1^H-NMR (DMSO-*d*_6_), δ ppm: 4.0 (S, 1H, –NH), 4.42 (S, 2H, N–CH_2_–N–), 5.16 (S, 2H, –OCH_2_), 7.06–8.04 (m, 13H, aromatic rings), ^13^C-NMR (CDCl_3_ ,δ ppm): 69.8, 70.8, 114.4, 116.3, 118.0, 125.9, 127.1, 127.6, 128.9, 130.2, 131.7, 135.6, 136.7, 146.7, 157.0, 161.3, 177.1, *m*/*z* 431.10 (100%), 435.11 (97.4%), 436.10 (4.5%), Molecular Formula: C_22_H_18_N_4_O_4_S. Elemental Analysis: Calculated (C, H, N) 60.82, 4.18, 12.90, Found: 60.78, 4.13, 12.86.

*5-(4-(Benzyloxy)phenyl)-3-(((2*,*4-dinitrophenyl)amino)methyl)-1*,*3*,*4-oxadiazole-2(3H)-thione* (**6i**): Yield: 82%; m.p.: 145 °C. IR (KBr) ν_max_ (cm^−1^): 3247 (NH), 3100–3000 aromatic CH stretch, 2870–2800 aliphatic CH stretch, 1621 (C=N), 1593 (C–C), 1524 and 1344 (NO_2_), 1426 (C=S), 1262 and 1083 (C–O–C), 1405–1418 (C–NO_2_), ^1^H-NMR (DMSO-*d*_6_), δ ppm: 4.0 (S, 1H, –NH), 4.42 (S, 2H, N–CH_2_–N–), 5.16 (S, 2H, –OCH_2_), 7.06–8.88 (m, 12H, aromatic rings), ^13^C-NMR (CDCl_3_, δ ppm): 69.8, 70.8, 114.4, 116.3, 120.8, 127.1, 127.6, 128.9, 130.2, 130.8, 135.2, 136.7, 137.2, 152.8, 157.0, 161.3, 177.1, *m*/*z* 479.09 (100%), 480.09 (26.7%), 481.09 (6.4%), Molecular Formula: C_22_H_17_N_5_O_6_S. Elemental Analysis: Calculated (C, H, N) 55.11, 3.57, 14.61, Found: 55.08, 3.52, 14.57.

*5-(4-(Benzyloxy)phenyl)-3-(morpholinomethyl)-1*,*3*,*4-oxadiazole-2(3H)-thione* (**6j**): Yield: 85%; m.p.: 200 °C. IR (KBr) ν_max_ (cm^−1^): 3100–3000 aromatic CH stretch, 2870–2800 aliphatic CH stretch, 1623 (C–N), 1592 (aromatic C–C), 1437 (C=S), 1222 and 1036 (C–O–C), 2916/2850 (CH_2_ of morpholine), ^1^H-NMR (CDCl_3_), δ ppm: 2.75 (t, 4H, N–CH_2_–C), 3.62 (t, 4H, O–CH_2_–C), 3.72 (S, 2H, N–CH_2_–N–), 5.16 (S, 2H, –OCH_2_), 7.06–8.02 (m, 9H, aromatic rings), ^13^C-NMR (CDCl_3_,δ ppm): 67.91, 68.23, 70.12, 115.47, 119.51, 125.68, 128.17, 128.43, 128.61, 129.00, 131.97, 135.64, 156.69,177.14 *m*/*z* 383.13 (100%), 384.13 (23.7%), 385.13 (5.6%), Molecular Formula: C_20_H_21_N_3_O_3_S. Elemental Analysis: Calculated (C, H, N) 2.64, 5.52, 10.96 Found: 62.61, 5.48, 10.92.

*5-(4-(Benzyloxy)phenyl)-3-((phenylamino)methyl)-1*,*3*,*4-oxadiazole-2(3H)-thione* (**6k**): Yield: 80%; m.p.: 80 °C. IR (KBr) ν_max_ (cm^−1^): 3553 (NH), 3100–3000 aromatic CH stretch, 2870–2800 aliphatic CH stretch, 1612 (C=N), 1595 (C–C), 1428 (C=S), 1255 and 1151 (C–O–C), ^1^H-NMR (CDCl_3_ + DMSO-*d*_6_), δ ppm: 4.0 (S, 1H, –NH), 4.42 (S, 2H, –N–CH–_2_N–), 5.16 (S, 2H, –OCH_2_), 6.77–8.02 (m, 14H, aromatic rings), ^13^C-NMR (CDCl_3_, δ ppm): 70.8 113.5, 114.4, 116.3, 120.8, 127.1, 127.6, 128.9, 129.5, 130.2, 147.6, 157.0, 161.3, 177.1, *m*/*z* 383.13 (100%), 384.13 (23.7%), 385.13 (5.6%). Molecular Formula: C_22_H_19_N_3_O_2_S. Elemental Analysis: Calculated (C, H, N) 67.84, 4.92, 10.79 Found: 67.79, 4.88, 10.74.

*5-(4-(Benzyloxy)phenyl)-3-(((4-methoxyphenyl)amino)methyl)-1*,*3*,*4-oxadiazole-2(3H)-thione* (**6l**): Yield: 81%; m.p.: 100 °C IR (KBr) ν_max_ (cm^−1^): 3550–3235 (NH), 3100–3000 aromatic CH stretch, 2870–2800 aliphatic CH stretch, 1615 (C=N), 1593 (C–C), 1425 (C=S), 1250 and 1152 (C–O–C), 1450 (C–OCH_3_), ^1^H-NMR (CDCl_3_ + DMSO-*d*_6_), δ ppm: 3.83 (S, 3H, –OCH_3_), 4.0 (S, 1H, –NH), 4.42 (S, 2H, N–CH_2_–N−), 5.16 (S, 2H, –OCH_2_),6.77–8.02 (m, 13H, aromatic rings), ^13^C-NMR (CDCl_3_, δ ppm): 55.8, 70.8, 114.4, 115.1, 115.8, 127.1, 127.6, 128.9, 130.2, 139.9, 151.7, 157.0, 161.3, 177.1, *m*/*z* 419.13 (100%), 420.13 (26.9%), 421.13 (5.6%). Molecular Formula: C_23_H_21_N_3_O_3_S. Elemental Analysis: Calculated (C, H, N) 65.85, 5.05, 10.02 Found: 65.81, 5.00, 10.00.

*4-(((5-(4-(Benzyloxy)phenyl)-2-thioxo-1*,*3*,*4-oxadiazol-3(2H)-yl)methyl)amino)benzoic acid* (**6m**): Yield: 79%; m.p.: 140 °C. IR (KBr) ν_max_ (cm^−1^): 3100–3000 aromatic CH stretch, 2870–2800 aliphatic CH stretch, 1674 (C=O), 1613 (C=N), 1606 (C–C), 1431 (C=S), 1269 and 1185 (C–O–C), 3550–3245 (NH and COOH), ^1^H-NMR (DMSO-*d*_6_), δ ppm: 4.0 (S, 1H, –NH), 4.42 (S, 2H, N–CH_2_–N–), 5.16 (S, 2H, –OCH_2_), 6.8–8.02 (m, 13H, aromatic rings), 10.50 (s, 1H, –COOH). ^13^C-NMR (CDCl_3_, δ ppm): 70.8, 112.0, 114.4, 116.3, 118.6, 127.1, 127.6, 128.9, 130.2, 131.1, 152.8, 157.0, 161.3, 169.3, 177.1, *m*/*z* 433.11 (100%), 434.11 (26.9%), 435.11 (5.8%). Molecular Formula: C_23_H_19_N_3_O_4_S. Elemental Analysis: Calculated (C, H, N) 63.73, 4.42, 9.69 Found: 63.67, 4.39, 9.62.

*5-(4-(Benzyloxy)phenyl)-3-((methylamino)methyl)-1*,*3*,*4-oxadiazole-2(3H)-thione* (**6n**):Yield: 78%; m.p.: 110 °C. IR (KBr) ν_max_ (cm^−1^): 3552 (NH), 3100–3000 aromatic CH stretch, 2870–2800 aliphatic CH stretch, 1615 (C=N), 1593 (C–C), 1425 (C=S), 1250 and 1152 (C–O–C), 1410 (N–CH_3_). ^1^H-NMR (DMSO-*d*_6_), δ ppm: 2.0 (s, 1H, NH), 3.26 (s, 3H, CH_3_), 3.91 (S, 2H, N–CH_2_–N–), 5.16 (S, 2H, –OCH_2_), 7.06–8.02 (m, 9H, aromatic rings), ^13^C-NMR (CDCl_3_, δ ppm) :26.0, 27.5, 70.8, 72.5, 114.5, 127.1, 127.6, 128.3, 128.4, 128.9, 129.3, 136.7, 189.8. *m*/*z* 327.10 (100%), 328.11 (18.7%), 329.10 (4.7%). Molecular Formula: C_17_H_17_N_3_O_2_S. Elemental Analysis: Calculated (C, H, N): 62.36, 5.23, 12.83 Found: 62.31, 5.19, 12.79.

*5-(4-(Benzyloxy)phenyl)-3-((biphenylamino)methyl)-1*,*3*,*4-oxadiazole-2(3H)-thione* (**6o**):Yield: 79%; m.p.: 115 °C. IR (KBr) ν_max_ (cm^−1^): 3100–3000 aromatic CH stretch, 2870–2800 aliphatic CH stretch, 1615 (C=N), 1593 (C–C), 1425 (C=S), 1250 and 1152 (C–O–C), 1300 (C–N sec. amine), ^1^H-NMR (DMSO-*d_6_*), δ ppm: 4.42 (S, 2H, N–CH_2_–N–), 5.16 (S, 2H, –OCH_2_), 6.77–8.02 (m, 19H, aromatic rings), ^13^C-NMR (CDCl_3_, δ ppm): 70.8, 80.0, 110.1, 114.4, 116.3, 119.1, 121.9, 127.6, 128.9, 129.6, 130.2, 136.7, 149.1, 157.0, 161.3, 177.1. *m*/*z* 465.15 (100%), 466.15 (32.2%), 467.15 (5.1%), Molecular Formula: C_28_H_23_N_3_O_2_S. Elemental Analysis: Calculated (C, H, N): 72.23, 4.98, 9.03, Found: 72.20, 4.92, 9.0.

### 3.6. Antifungal Screening

*In vitro* antifungal susceptibility testing was performed by the broth micro dilution method against seven human pathogens:*C. albicans* ATCC 24433, *C. albicans* ATCC 10231, *Candida glabrata* NCYC 388, *Cryptococcus neoformans* ATCC 34664, *Cryptococcus neoformans* PRL 518, *Aspergillus fumigatus* NCIM 902, *Aspergillus niger* ATCC 10578 following the Clinical Laboratory Standards Institute protocols M27-A3 (for yeasts) and M38-A2 (for filamentous fungi) [31,32,33,34]. Briefly, appropriate amount of compounds were dissolved in dimethyl sulfoxide to get 100× final strength. The stock was then diluted 1:50 in RPMI 1640 medium and 200 μL of this solution was added to the first row of a 96-well microtitre plate. The compounds were serially diluted two-fold in successive wells to get a range of 2–256 μg/mL. Spores of the filamentous fungi (~2 × 10^4^ spores/mL) and yeast cells freshly grown in YPG broth in logarithmic phase (~2 × 10^3^ cfu/mL) were suspended in the RPMI 1640 medium and 100 μL from these were inoculated in the wells of the plate. The micro titer plates were incubated for 24–48 h. Growth was checked by visual observation and measuring absorbance at 600 nm using micro titer plate reader. The IC_50_ was defined as the concentration exhibiting 50% inhibition of the growth as compared to the growth of control.

### 3.7. Molecular Docking Data

**Figure 2:**
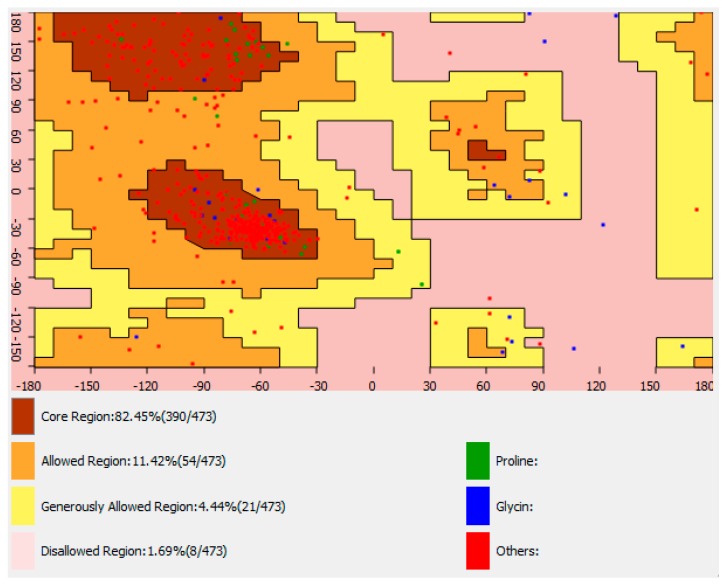
Ramachandran Plot for structural validation.

The 3D model structure of cytochrome P450 lanosterol 14α-demethylase of *C. albicans* was built using homology modeling [3,33]. The amino acid sequence of the enzyme was obtained from the Universal Protein Resource (http://www.uniprot.org/, Accession Code: P10613) and sequence homologs were obtained from Protein Data Bank (PDB) using Blast search. Based on the Blast search result, we used the crystal structure of human lanosterol 14α-demethylase (CYP51) with azole as a template for homology modeling (PDB ID: 3LD6). The VLifeMDS 4.3 ProModel was used for modeling of the 3D structure of protein based on the amino acid sequences of a close homologue. Alignment of amino acid sequence of CA-CYP51 (P10613) and human CYP51 (3LD6_B) is shown in Figure S1. The Blosum-62 matrix was used with a gap penalty of 1. The model was then energy minimized using the MMFF94 force field [35]. Manual inspection was made to ensure the conserved motifs and loops were correctly aligned. The quality of generated *C. albicans* lanosterol 14α-demethylase model was assessed by using a well validated program like PROCHECK [36]. The structural validation was performed by using Ramachandran plot and is shown in Figure 2. The further structural super imposition was performed to know the structural coordinate of target protein and RMSD value was found within standard range of 0.997607 Å. The molecular docking study of the synthesized compounds **6a**–**o** and the standard drugs fluconazole and miconazole were performed against homology built cytochrome P450 lanosterol 14α-demethylase of *C. albicans* to understand the binding interactions using VLife MDS 4.3 package following standard procedures [37].

## 4. Conclusions

Fifteen novel 5-(4-(benzyloxy) phenyl)-3-((phenylamino)methyl)-1,3,4-oxadiazole-2(3*H*)-thione derivatives were successfully synthesized using molecular sieves under ultrasound irradiation giving better yields of 78%–90% after shorter reaction times of 1–2 h in contrast to conventional reactions which require 15–20 h refluxing. Compounds **6c**, **6f** and **6i** exhibited promising antifungal activity and the developed scaffold offers an attractive template for lead antifungal discovery.

## Figures and Tables

**Table 1:** Comparison of reaction kinetics of conventional and ultrasonic irradiation methods for the synthesized compounds **6a**–**o**.

Entry	Conventional		Ultrasonic Irradiation	
	Time (h)	Yield (%)	Time (h)	Yield (%)
**6a**	15	68	1.5	80
**6b**	16	63	1.0	81
**6c**	15	62	1.5	78
**6d**	17	58	2.0	79
**6e**	18	45	1.0	81
**6f**	18	56	1.0	79
**6g**	19	57	2.0	81
**6h**	17	61	1.5	90
**6i**	18	53	1.0	82
**6j**	17	63	1.5	85
**6k**	15	58	1.5	80
**6l**	17	57	1.0	81
**6m**	15	61	2.0	79
**6n**	16	58	2.0	79
**6o**	15	45	2.0	78

**Table 2:** Antifungal activity of the compounds **6a**–**o** (IC_50_ * in μg/mL) against different human pathogenic fungi.

Compound	*Candida albicans* ATCC 24433	*Candida albicans* ATCC 10231	*Candida glabrata* NCYC 388	*Cryptococcus neoformans* ATCC 34664	*Cryptococcus neoformans* PRL 518	*Aspergillus fumigatus* NCIM 902	*Aspergillus niger* ATCC 10578
**6a**	64	49.6	64	64	64	>256	**27.7**
**6b**	128	67.12	59.6	77.5	>128	**24.2**	**24.1**
**6c**	21.6	108	53.6	108.9	196.5	**11.2**	93
**6d**	68	159	65.6	128	>128	**6**	130
**6e**	54.4	44.6	**24.5**	64	**14.21**	**25.8**	**47**
**6f**	**16**	84.9	**5.4**	>256	90	256	256
**6g**	62	107.6	256	76.8	94.7	**4**	>256
**6h**	55.3	57.4	64	47.6	66.6	**16**	**18.61**
**6i**	23.3	80.6	40.3	256	93.5	**24.6**	>256
**6j**	38.4	31.5	56.6	192	39	**8**	128
**6k**	150	53.3	36.2	64.2	146.6	35.1	**22.9**
**6l**	115	95.3	42.5	49.7	145.8	81	73.2
**6m**	91.5	49.7	78.6	62.5	54.6	87.4	84.8
**6n**	30.3	43	48.8	47.5	105.6	81.5	56.9
**6o**	95	**28.1**	50.7	**42.7**	156.5	76.1	70.5
**Fluconazole**	0.12	0.11	9.4	16	4	64	46

* IC_50_ is the concentration exhibiting 50% inhibition of the growth as compared to the growth of control.

**Table 3:** Results of the docking study of the compounds.

Compound	Binding Energy	Hydrogen Bonds	Hydrophobic Bonds
**6a**	−41.69	LEU412-O=C	ALA343, GLY344, LEU412, MET415, PHE499, GLY508 and ALA512
**6b**	−49.33	LEU412-O=C	ALA343, THR347, MET415, GLY500 and HIS504
**6c**	**−57.85**	CYS506-O-CH_2_	LEU412, SER414, MET415, CYC506, ILE507 and GLY508
**6d**	−45.23	CYS506-O-CH_2_	ALA343, LEU412, MET415, CYC506, ILE507 and GLY508
**6e**	−35.50	LEU412-O=C; GLY500-N of nitro and GLY500-O-N of NO_2_	ALA343, LEU412, ILE507 and GLY508
**6f**	**−65.25**	CYS506-NH	ALA343, THR347, MET415, PHE499, HIS504, CYC506, GLY508, GLU509, ALA512 and TYR513
**6g**	−52.37	LEU412-O=C	THR347, LEU412, MET415, PHE499 and HIS504
**6h**	−47.18	VAL497 and GLY500-N of NO_2_	ALA343, GLY344, PHE499, GLY500 and GLY508
**6i**	**−61.03**	TYR154-O-N of 2-nitro; TYR168- O=C and MET415-N of 4-nitro	LEU340 and ALA343
**6j**	−40.29	LEU412-O-CH2	ALA343, GLY344, LEU412, MET415, PHE499, GLY508 and ALA512
**6k**	−37.85	-	ALA343, GLY344, THR347, PHE499, GLY500 and GLY508
**6l**	−42.91	-	LEU186, LEU240, GLY344, THR347, MET415, PHE499, GLY500, CYS506, GLY508 and PHE511
**6m**	−36.83	-	ALA343, LEU412 and PHE499
**6n**	−40.09	CYS506-O-CH_2_	ALA343, VAL440, PRO442, VAL497, GLY500 and CYC506
**6o**	−38.97	TYR168-O=C	LEU340, ALA343, ILE507 and GLY508
Fluconazole	−67.29	TYR168-F of phenyl	GLY500 and HIS504

## Supplementary Materials

Supplementary materials can be accessed at: http://www.mdpi.com/1420-3049/21/5/484/s1.
