# Supplementary material for: Ultrasound- and Molecular Sieves-Assisted Synthesis, Molecular Docking and Antifungal Evaluation of 5-(4-(Benzyloxy)-substituted phenyl)-3-((phenylamino)methyl)-1,3,4-oxadiazole-2(3H)-thiones
Source: Molecules. 2016 May 10;21(5):484. doi: 10.3390/molecules21050484 (PMC6273549; doi:10.3390/molecules21050484)
Supplement: Supplementary file 1 [file molecules-21-00484-s001.pdf]

Supplementary Materials: Ultrasound- and Molecular Sieves-Assisted Synthesis, Molecular Docking and Antifungal Evaluation of 5-(4-(Benzyloxy)-substituted phenyl)-3-((phenylamino)methyl)-1,3,4-oxadiazole-2(3*H*)-thiones

Urja D. Nimbalkar, Santosh G. Tupe, Julio A. Seijas Vazquez, Firoz A. Kalam Khan, Jaiprakash N. Sangshetti and Anna Pratima G. Nikalje

Table S1. Characterization data of Mannich bases (**6a**–**o**).

Derivatives	R/Ar	(Mol. Wt)	Yield (%)	m.p. (°C)	R_f
**a**	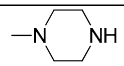	382.48	80	205	0.4
**b**	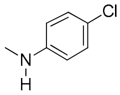	423.92	81	180	0.7
**c**	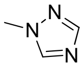	365.41	78	235	0.5
**d**	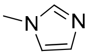	364.42	79	240	0.4
**e**	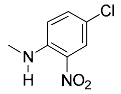	468.91	81	218	0.6
**f**	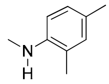	417.52	79	230	0.4
**g**	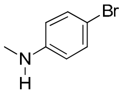	468.37	81	228	0.6
**h**	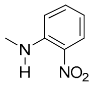	434.47	90	220	0.3
**i**	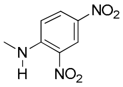	479.47	82	145	0.5
**j**	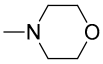	383.46	85	200	0.4
**k**	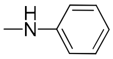	389.47	80	80	0.3
**l**	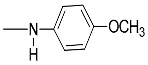	419.13	81	100	0.5
**m**	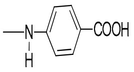	433.48	79	140	0.5
**n**	—HN−CH_3	327.40	78	110	0.2
**o**	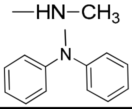	465.57	79	115	0.4

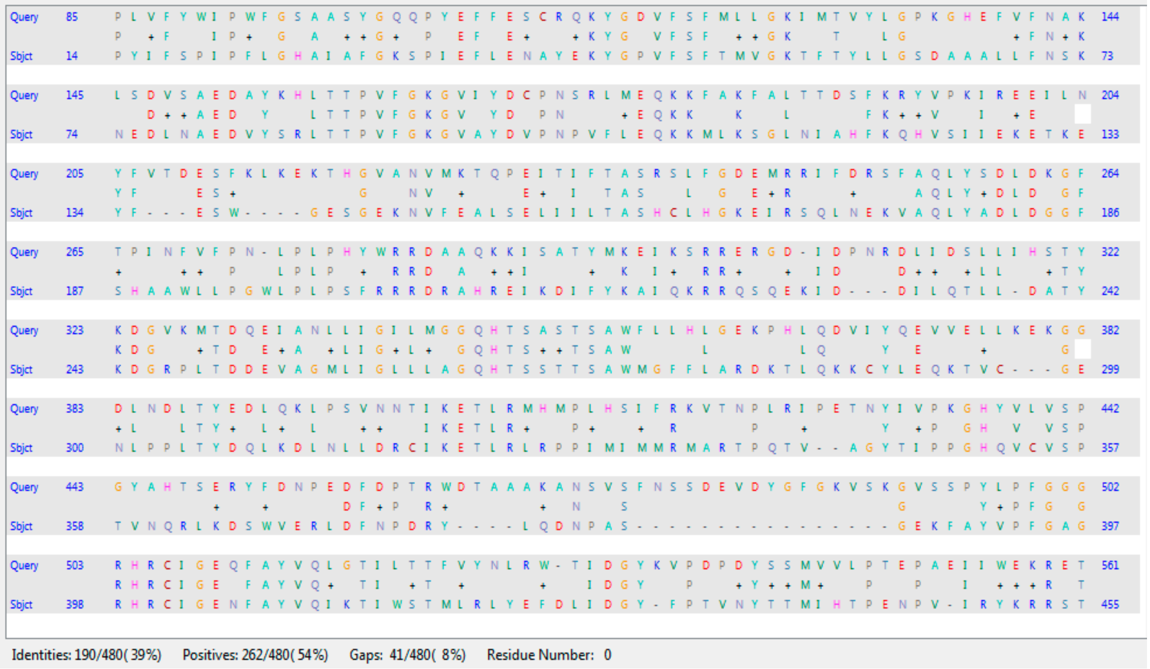

Figure S1. Alignment of amino acid sequence of CA-CYP51 (P10613) and human CYP51 (3LD6_B).
